# Supplementary material for: Targeting ALK averts ribonuclease 1-induced immunosuppression and enhances antitumor immunity in hepatocellular carcinoma
Source: Nat Commun. 2024 Feb 2;15:1009. doi: 10.1038/s41467-024-45215-0 (PMC10837126; doi:10.1038/s41467-024-45215-0)
Supplement: Supplementary file 1 — Supplementary Information [file 41467_2024_45215_MOESM1_ESM.pdf]

Targeting ALK averts ribonuclease 1-induced immunosuppression and enhances anti-tumor immunity in hepatocellular carcinoma

Chunxiao Liu et. al

Supplementary Figures and legends

Supplementary Tables

Supplementary Figure 1

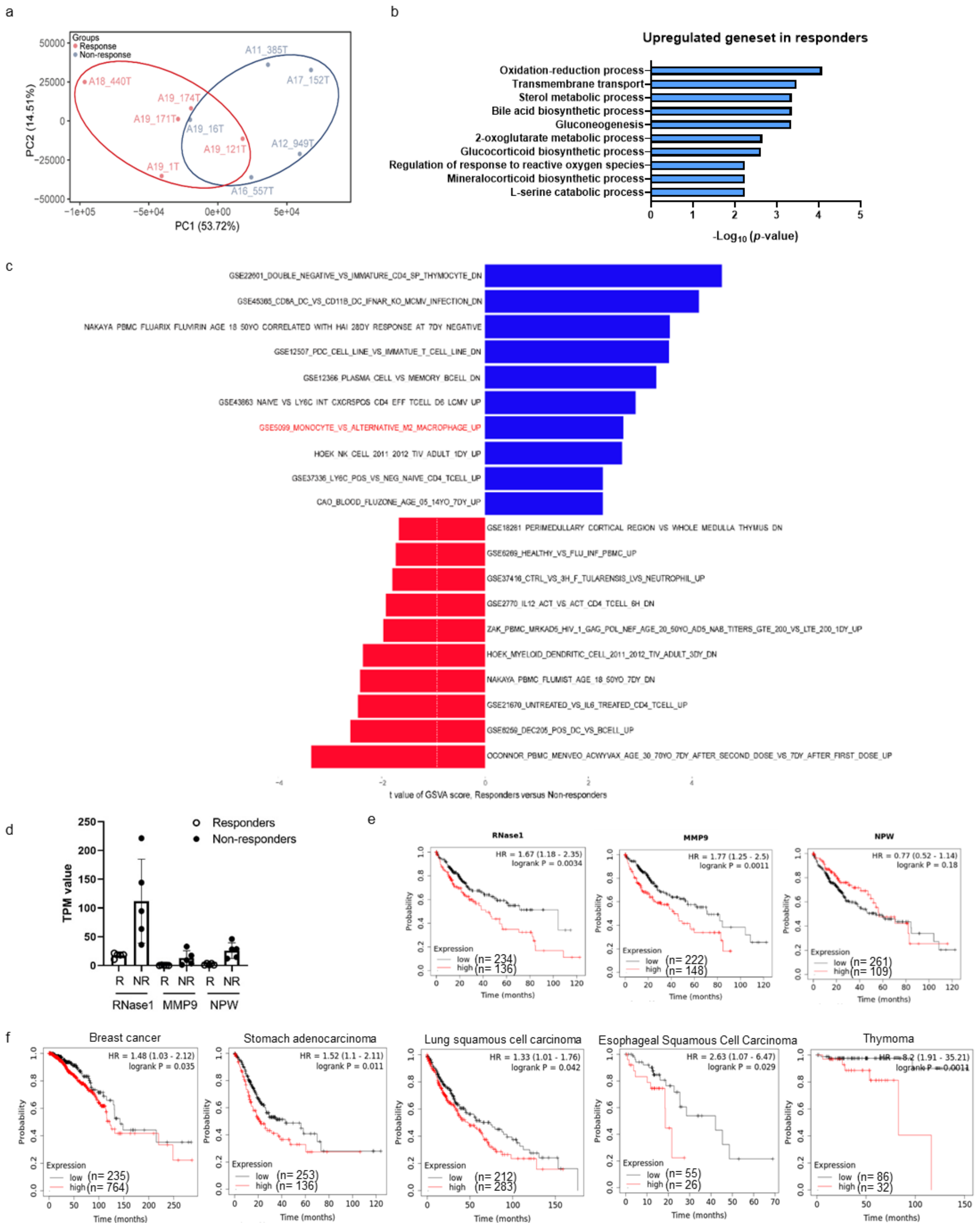

g

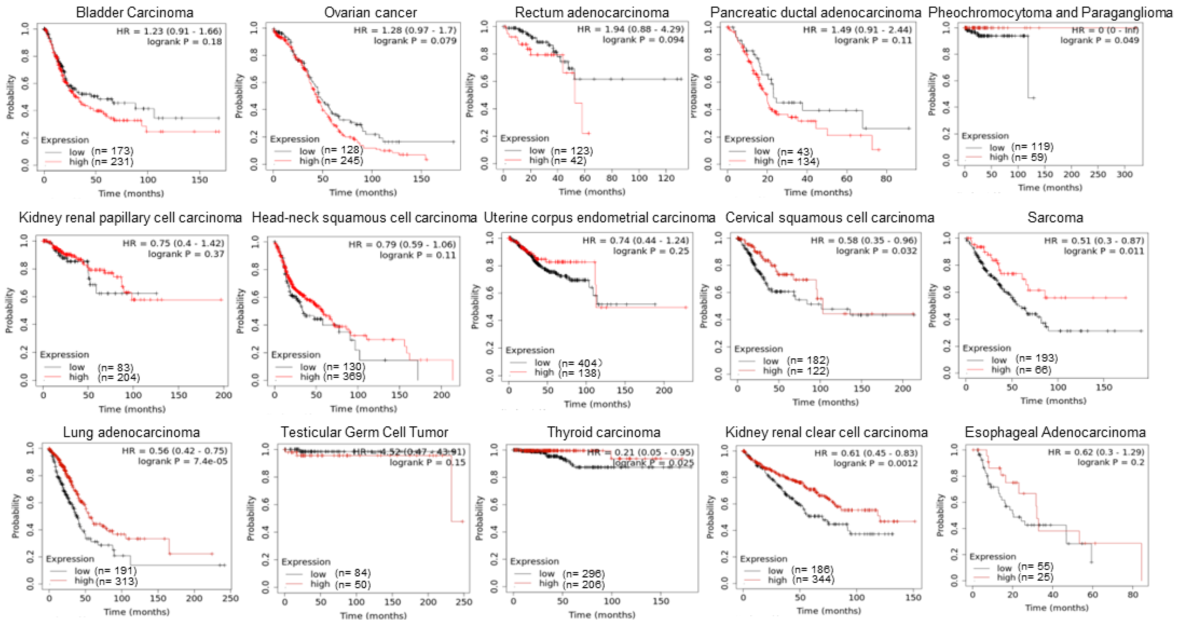

h

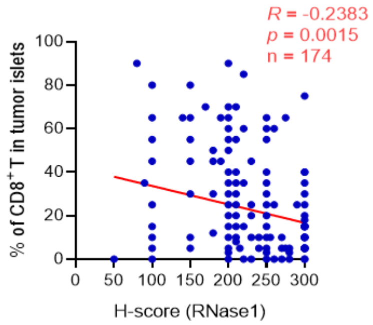

Supplementary Figure 1 | GO analysis of RNA sequencing data and correlation between RNase1 expression and OS rate for human cancers. a, Principal component analysis of 10 samples from anti-PD-1 therapy responders (n = 5) and nonresponders (n = 5). Each point in the plot represents one sample and is color-coded according to clinical response to nivolumab. Responder (red) and nonresponder (blue) samples clustered separately according to the first two principal component scores (PC1 and PC2) are shown. **b,** The 10 most frequautly enriched biological process GO terms in nivolumab and responders. Upregulated genes in each group are functionally annotated using GO terminology, and the top pathways with statistical significance are shown. **c,** GSVA analysis between responders and non-responders by setting C7:

immunologic signatures as reference gene sets. Top 10 enriched immunologic pathways in responders and non-responders were shown. **d,** TPM value of RNase1, MMP9, and NPW genes from RNAseq analyses for HCC patients receiving anti-PD-1 treatment. **e,** Kaplan-Meier estimates of OS of liver cancer in relation to RNase1, MMP9, and NPW mRNA expression. **f and g,** Kaplan-Meier estimates of OS of Pan-cancer in relation to RNase1 mRNA expression. **h,** Negative correlation between RNase1 and CD8+ T cell infiltration in HCC patients (n = 174; R = -0.24 [Pearson's chi-square test]).

Supplementary Figure 2

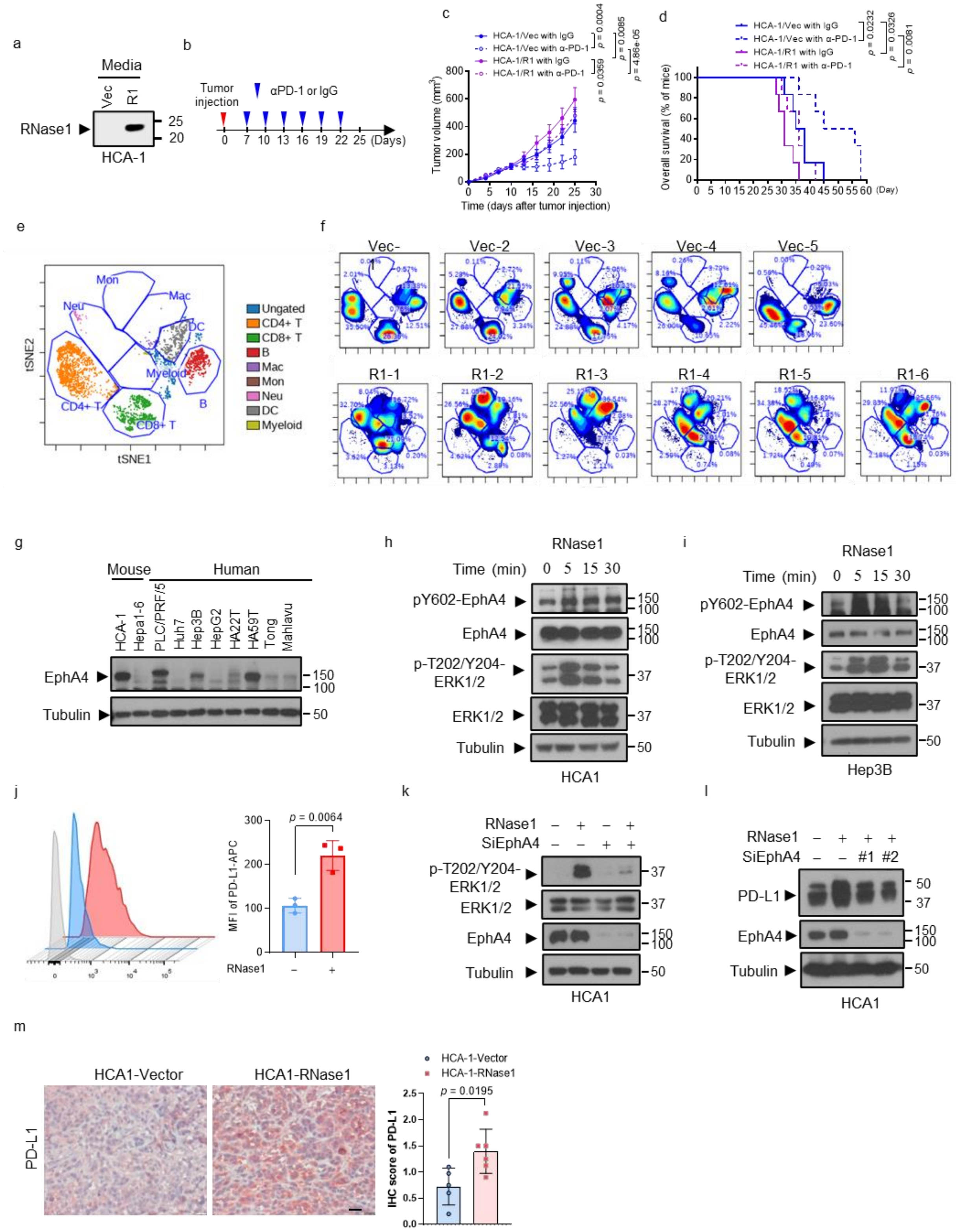

Supplementary Figure 2 | Positive correlation of RNase1 expression with immunosuppression and PD-L1 expression. a, Immunoblot of secreted RNase1 in HCA-1 cells with stable ectopic RNase1 expression. **b,** Schematic of the protocol for anti-PD-1 (αPD-1) and IgG-based treatment in the subcutaneous HCC model. Stable HCA-1/Vec or HCA-1/R1 cells were injected into C3H mice on day 0, and αPD-1 (100 μg per mouse) or IgG was administered via intraperitoneal injection starting on day 7 and continuing for 15 days. **c,** Tumor growth after injection of the indicated cells in C3H mice given αPD-1 or IgG (*n* = 6 mice per group; two-way ANOVA.). **d,** Survival of mice bearing HCA-1/Vec– or HCA-1/R1–derived tumors following treatment with αPD-1 or IgG (*n* = 6 mice per group; log-rank test). **e and f,** Immune cell populations identified using viSNE (**e**) in individual samples of HCA-1/Vec (Vec-1 to Vec-5) and HCA-1/R1 (R1-1 to R1-6) tumors (**f**), with cell populations defined based on basic phenotypic markers (Figure 2g). **g,** Expression of EphA4 in HCC cells. **h and i,** Immunoblots of HCA-1 (**h**) and Hep3B (**i**) cells treated with RNase1 (1 μg/ml) at the indicated time points. **j,** HCA-1 cells were treated with or without RNase1 and were stained with anti-PD-L1 antibody to determine the surface expression of PD-L1. The median fluorescence intensity (MFI) of PD-L1 of cells was quantified by flow cytometry. (n = 3; grey square: negative control (unstained HCA-1 cells); blue square: without RNase1 treatment; red square: with RNase1 treatment. The error bars represent mean (± SD) values. **k,** Immunoblot of HCA-1 cells transfected with EphA4 siRNAs (siEphA4) for 48 h following treatment with RNase1 (1 μg/ml) for 15 min. **l,** Immunoblot of HCA-1 cells transfected with siEphA4 in the presence or absence of RNase1 (1 μg/ml) for 24 h. **m,** Left, immunohistochemical stains of HCA-1/Vec and HCA-1/R1 tumor sections from mice with an anti-PD-L1 antibody. Scale bar, 50 μm. Right, quantification of immunohistochemistry scores for PD-L1 (*n* = 5 for HCA-1/Vec, *n* = 6 for HCA-1/R1). (**a**, **g**, **h**, **i**,

k, and **l**) Representative results from 3 independent experiments. Statistical analysis: **j** and **m,** two-sided Unpaired Student's t-test. Source data are provided as a Source Data file.

Supplementary Figure 3

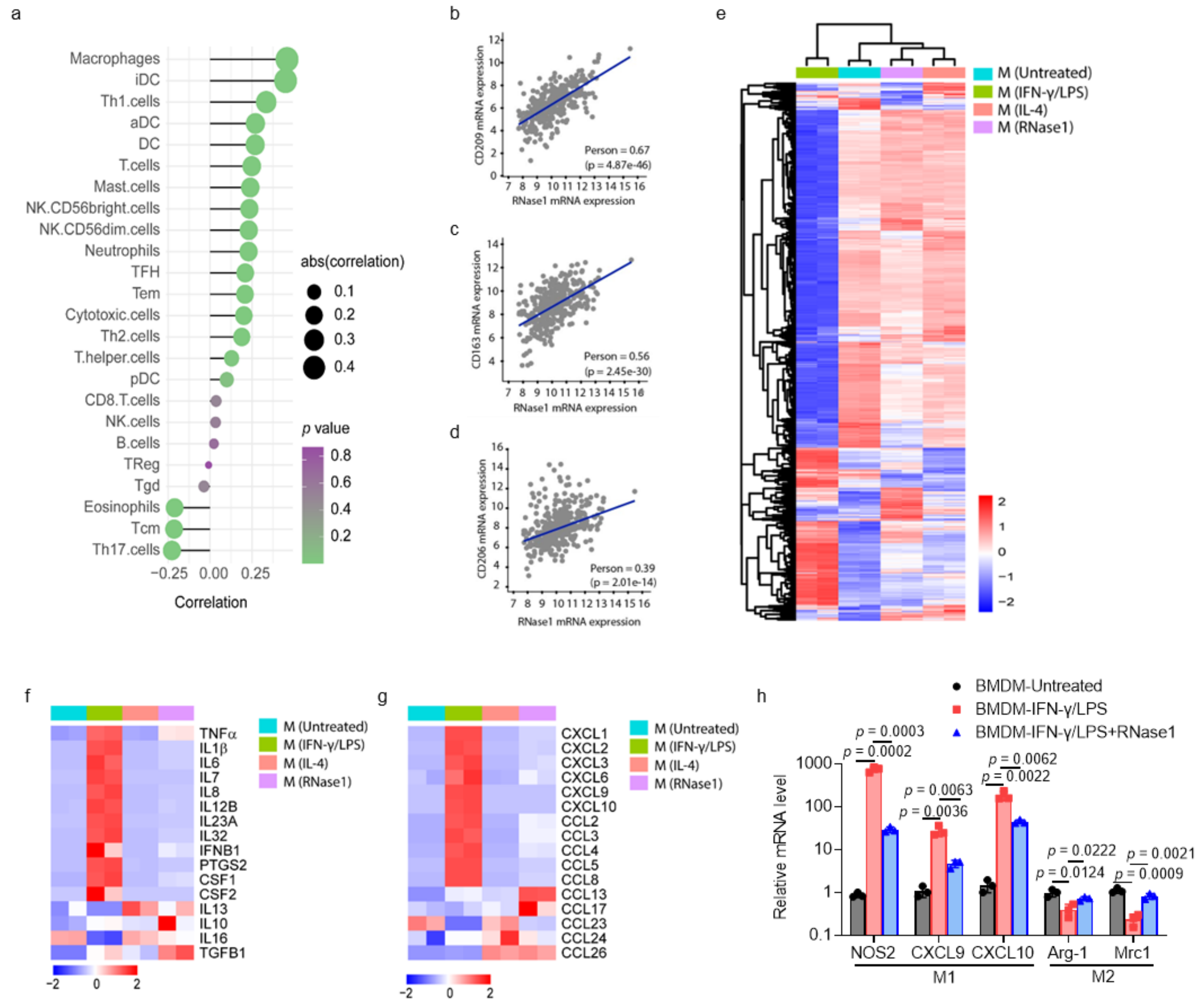

Supplementary Figure 3 | Correlation of RNase1 expression with M2-like polarized macrophage infiltration. a, Correlation of RNase1 expression with infiltrating immune cells. RNase1 expression data for HCC cases (n = 356). The size of each dot represents the strength of the correlation between RNase1 expression and immune cells. The color of each dot represents the P value. **b-d,** Pearson correlation of RNase1 expression with CD209 (**b**), CD163 (**c**), and

CD206 (**d**) expression (*n* = 356). **a-d**, Data were collected from TCGA HCC public data sets (http://cancergenome.nih.gov/). **e,** Heat map of the expression patterns for transcriptome-wide, differentially expressed transcription factor genes in differentiated THP-1 macrophages under no treatment or treatment with IFN-γ (10 ng/ml) plus LPS (200 ng/ml), IL-4 (20 ng/ml), or RNase1 (1 μg/ml) for 6 h (*n* = 2 samples per group). **f and g,** Heat maps of differentially expressed cytokines (**f**) and chemokines (**g**) in treatment with IFN-γ plus LPS, IL-4, or RNase1. **h,** The levels of mRNA expression for M1 and M2 markers in BMDMs. The BMDMs were exposed to IFN-γ (10 ng/ml) plus LPS (200 ng/ml) in the presence or absence of RNase1 (1 μg/ml) for 6 h. The mRNA expression for NOS2, CXCL9, and CXCL10 (M1 markers) and for Arg-1 and Mrc1 (M2 markers) was evaluated directly after incubation using quantitative reverse transcription-polymerase chain reaction (*n* = 3). Two-sided Unpaired Student's t-test. Source data are provided as a Source Data file.

Supplementary Figure 4

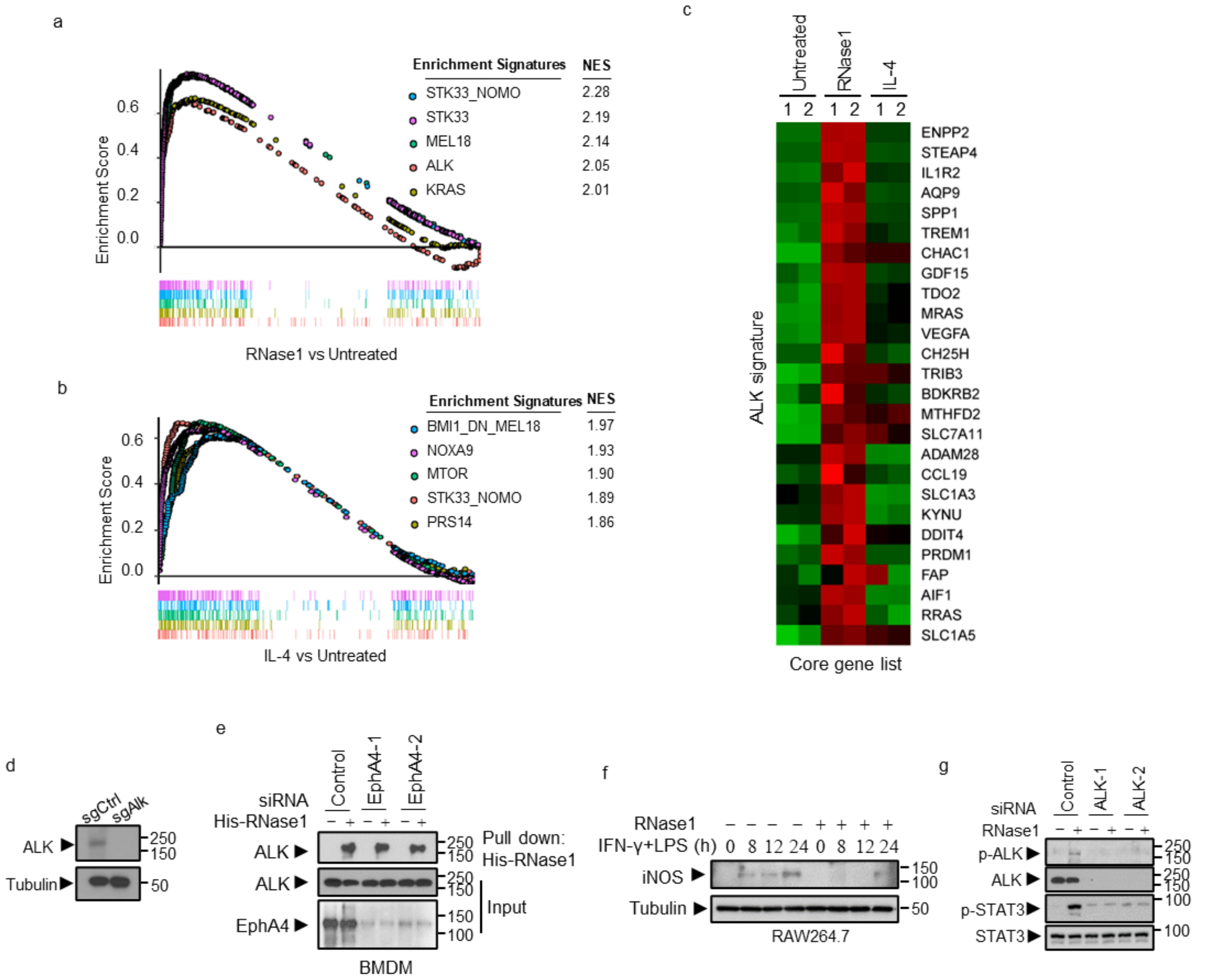

Supplementary Figure 4 | RNase1 binds to and activates ALK signaling. a and b, Graphs of the five most frequently enriched pathways in macrophages treated with RNase1 or IL-4. GSEA of THP-1–derived macrophages were performed after treatment with RNase1 (1 μg/ml; **a**) or IL-4 (20 ng/ml; **b**) for 6 h (n=2 samples per group). The normalized enrichment score (NES) is shown for each gene set. **c,** Heat map of gene sets of the ALK signature in each treatment group (n=2 samples per group). Only the genes identified as contributing to the enrichment score are shown. **d,** Western blot analysis of ALK expression in Raw264.7 sgCtrl or sgAlk cells. **e,** Pull-down assay and Western blot analysis of the interaction between RNase1 and ALK in BMDMs.

BMDMs were transfected with individual EphA4 siRNAs in the presence or absence of RNase1 (1 μg/ml). **f,** Western blot of Raw264.7 cells treated with IFN-γ (10 ng/ml) plus LPS (200 ng/ml) for various time points in the presence and absence of RNase1 (1 μg/ml). **g,** Immunoblot of BMDMs transfected with individual ALK siRNAs in the presence or absence of RNase1 (1 μg/ml) for 15 min. (**d-g**) Representative results from 3 independent experiments. Source data are provided as a Source Data file.

Supplementary Figure 5

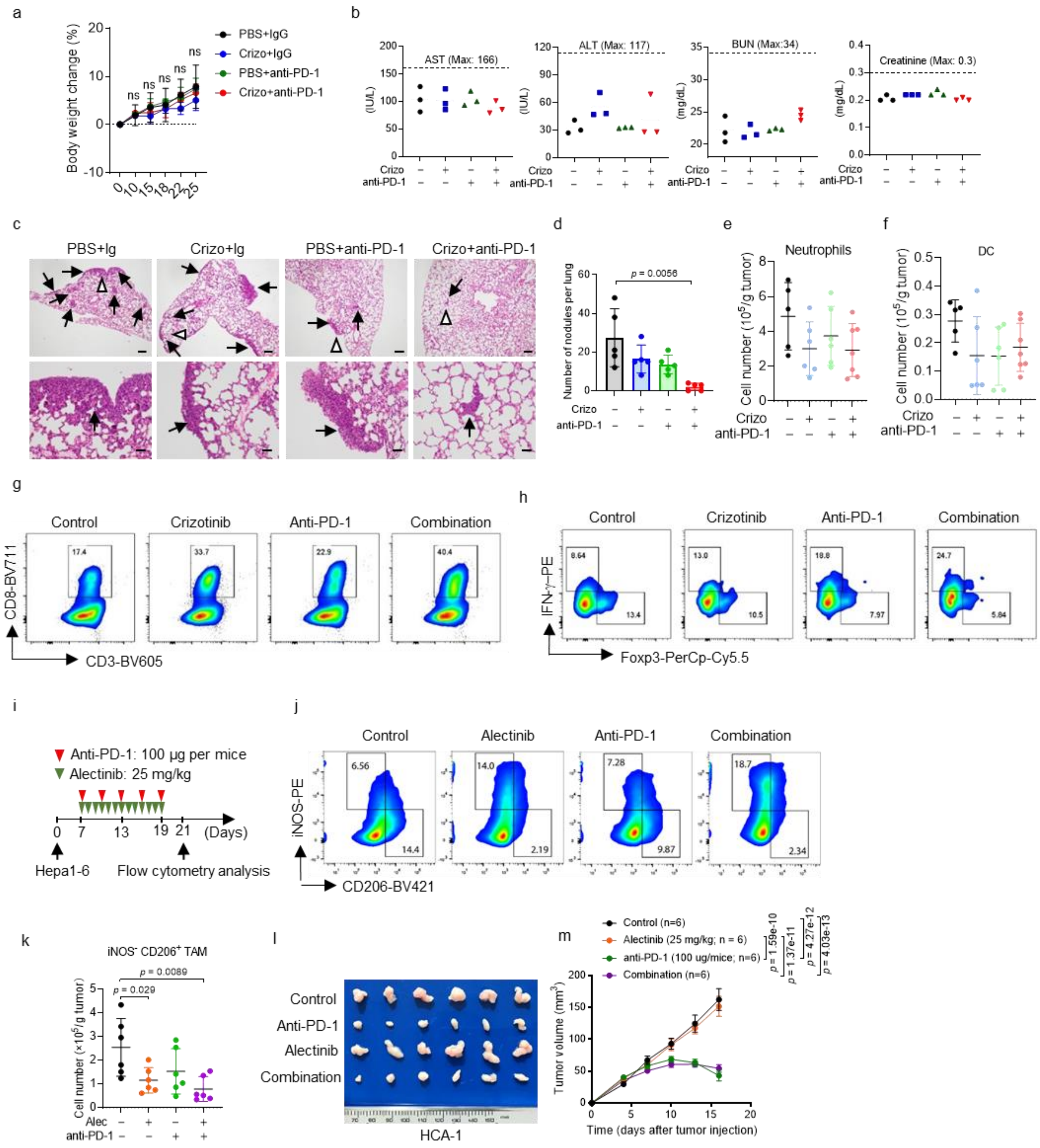

Supplementary Figure 5 | Combination therapy with an ALKi and anti-PD-1 antibody impeded HCC growth. a, Body-weight changes in mice with HCA-1/R1-derived tumors after each indicated treatment (n = 6 per group). The results were analyzed using two-way ANOVA. ns, not significant; PBS, phosphate-buffered saline; Crizo, crizotinib. **b,** Plots of liver and kidney

function indicators in mice given crizotinib, an anti-PD-1 antibody, or both (*n* = 3 per group). The normal ranges are indicated by the dashed lines. AST, aspartate aminotransferase; ALT, alanine transaminase; BUN, blood urea nitrogen. **c,** Representative hematoxylin- and eosin-stained images of lung tissue samples. Scale bar, 200 μm (top panels) and 50 μm (lower panels). The arrows indicate lung-metastatic nodules, and the labeled areas in the upper panel are shown in the lower panel at higher magnification. **d,** Bar graph depicting the number of metastatic nodules in the lungs in the indicated groups (*n* = 5 per group). **e and f,** Cell numbers of Neutrophils (**e**) and DCs (**f**) in tumors from mice with indicated treatment. **g,** Representative percentages of $CD8^+$ T cells in HCA-1/R1-derived tumors after the indicated treatments. **h,** Representative percentages of Th1 cells (IFN-$\gamma^+$$Foxp3^-$) and Tregs (IFN-$\gamma^-$$Foxp3^+$) in HCA-1/R1-derived tumors after the indicated treatments. For e-h： *n* = 5 for control group; *n* = 6 for anti-PD-1 or Crizotinib treatment group; and n = 7 for combination group. **i,** Schematic of the protocol for combination treatment with an anti-PD-1 antibody and the ALKi Alectinib using Hepa1-6 HCC subcutaneous mouse model. Hepa1-6 cells were injected into C57B/6 mice on day 0, and the mice were administered a vehicle control (IgG or phosphate-buffered saline), Alectinib, αPD-1, or a combination of Alectinib and αPD-1 as indicated. **j,** Representative results of the percentages of $iNOS^-CD206^+$ and $iNOS^+CD206^-$ TAM subsets in the tumors of mice given alectinib, αPD-1, or both (*n* = 6 mice per group). **k,** Absolute numbers $iNOS^-CD206^+$ TAM subsets in the tumors of mice given alectinib, αPD-1, or both (*n* = 6 mice per group). **l and m**, representative images of mouse tumors (**l**) and tumor volume (**m**) of the indicated mice groups. The error bars represent mean (± SD) values. Statistical analysis: (**b**, **d**, **e**, **f**, and **k**)**,** two-sided Unpaired Student's t-test; a and m, two-way ANOVA. Source data are provided as a Source Data file.

Supplementary Figure 6

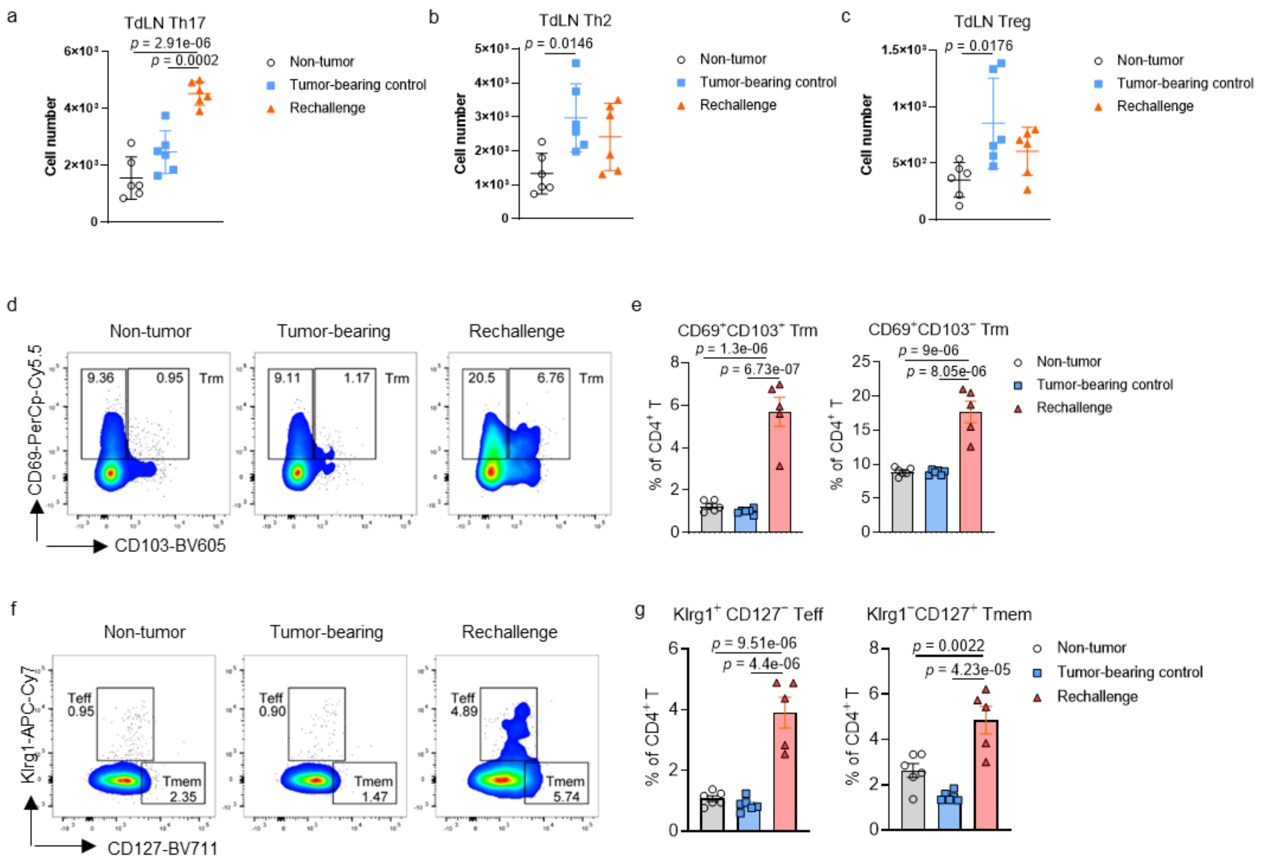

Supplementary Figure 6 | Combination therapy with an ALKi and anti-PD-1 antibody promoted Tmem-cell generation. a-c, Absolute numbers of Th17 cells (**a**), Th2 cells (**b**), and Tregs (**c**) in TdLNs from tumor-free, tumor-bearing control, and rechallenged mice (*n* = 6 per group). **d and e,** Percentages (**d**) and absolute numbers (**e**) of CD69^{+}CD103^{+} and CD69^{+}CD103^{-} Trm cells in TdLNs. **f and g,** Percentages (**f**) and absolute numbers (**g**) of Klrg1^{+}CD127^{-} subsets of CD4^{+} Teff cells and Klrg1^{-}CD127^{+} subsets of CD4^{+} Tmem cells in TdLNs. **d-g**: n=6 for non-tumor and tumor-bearing control groups; n=5 for rechallenge group. Statistical analysis: (**a**, **b**, **c**, **e**, and **g**), two-sided Unpaired Student's t-test. Source data are provided as a Source Data file.

Supplementary Figure 7

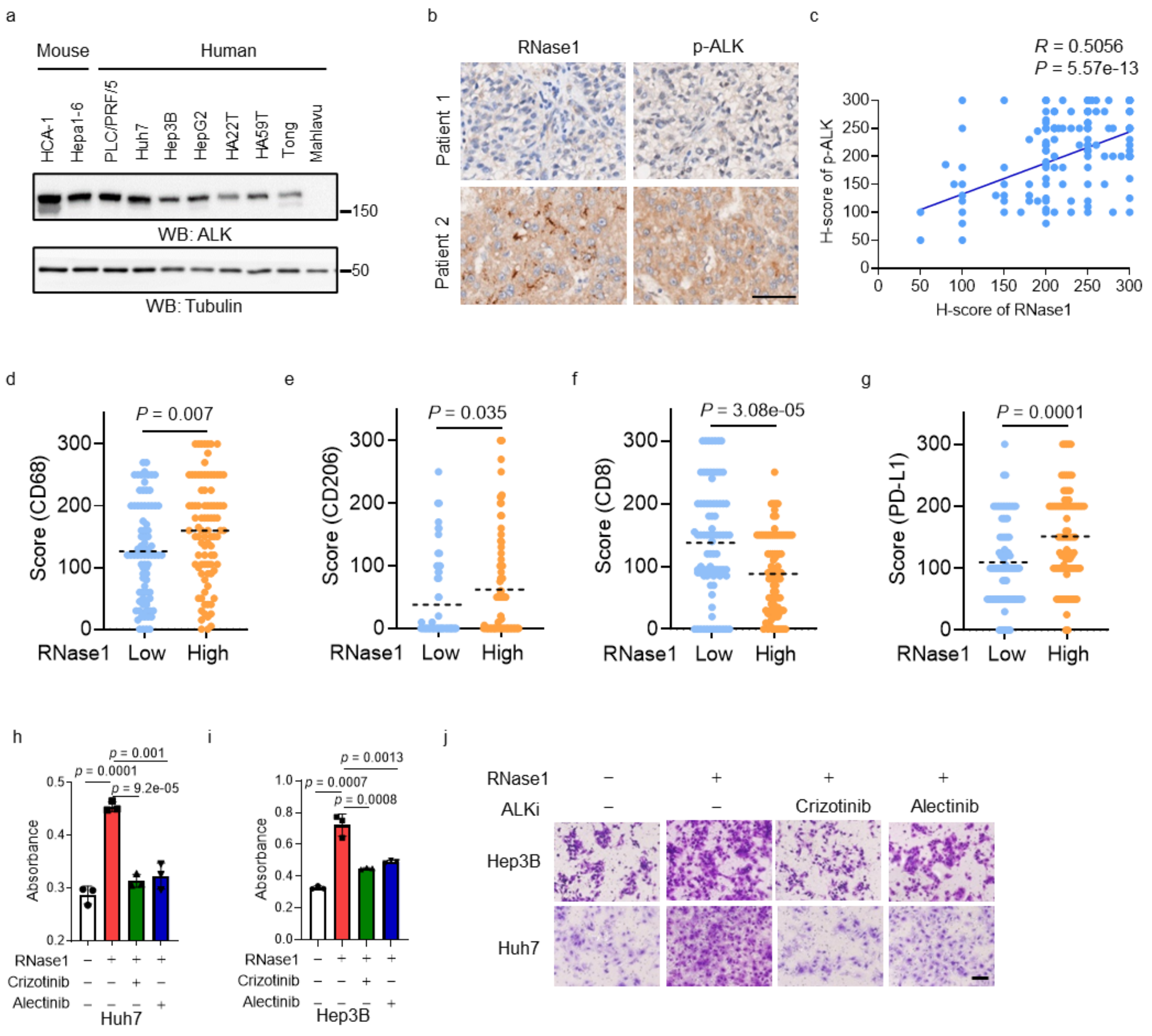

Supplementary Figure 7 | RNase1 expression is positively correlated with phospho-ALK and immunosuppression in HCC patients. a, Expression of ALK in HCC cells. **b,** Representative images of immunohistochemical staining for RNase1 and phospho-ALK expression in two HCC patients using an HCC tissue microarray. Scale bar, 50 μm. **c,** Positive correlation between RNase1 and phospho-ALK expression in HCC patients (n = 174; R = 0.5 [Pearson's chi-square test]). **d-g,** Relationship between RNase1 expression and the immunohistochemistry scores for CD68 (**d**), CD206 (**e**), CD8 (**f**), and PD-L1 (**g**) in an HCC

tissue microarray (*n* = 84 and *n* = 90 for the RNase1-low and -high groups, respectively; unpaired Student *t*-test). **h and i**, Cell viability of Huh7 (**h**) and Hep3B (**i**) cells with or without RNase1 (1g/ml) or ALK inhibitors treatment. **j**, representative images of cell invasion assay of Hep3B and Huh7 cells with indicated treatment. **h-j**: n=3 samples per group. Scale bar, 200 μm. (**a** and **j**) Representative results from 3 independent experiments. Statistical analysis: (**d**, **e**, **f**, **g**, **h** and **i**), two-sided Unpaired Student's t-test. Source data are provided as a Source Data file.

Supplementary Figure 8

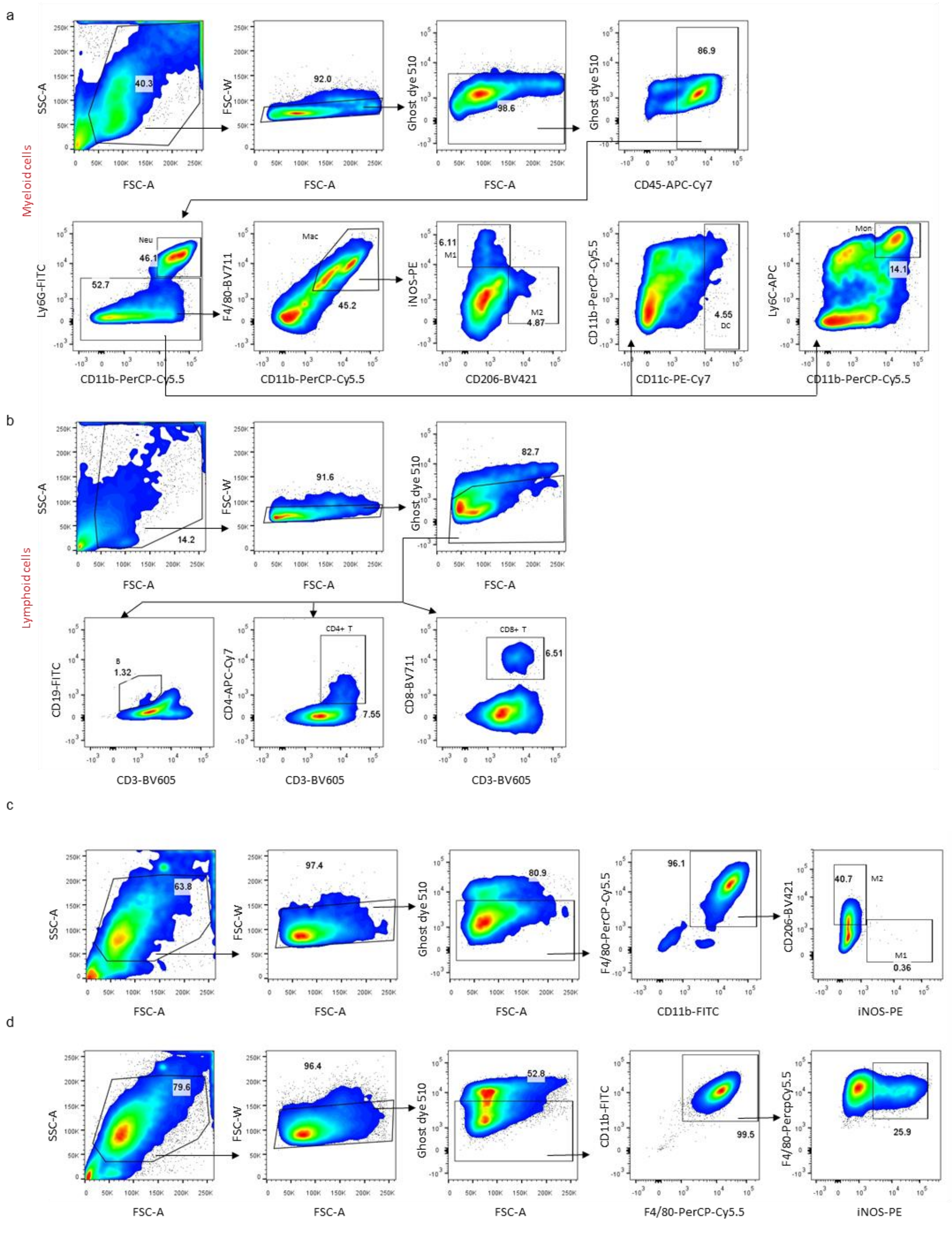

Supplementary Figure 8 | Flow cytometry gating strategy. a and b, Tumor gating strategy for myeloid cells (**a**) and lymphoid cells (**b**) in Figure 2 l-p. **c,** BMDM gating strategy for Figure 3d and 3e. **d,** BMDM gating strategy for Figure 4i.

Supplementary Figure 9

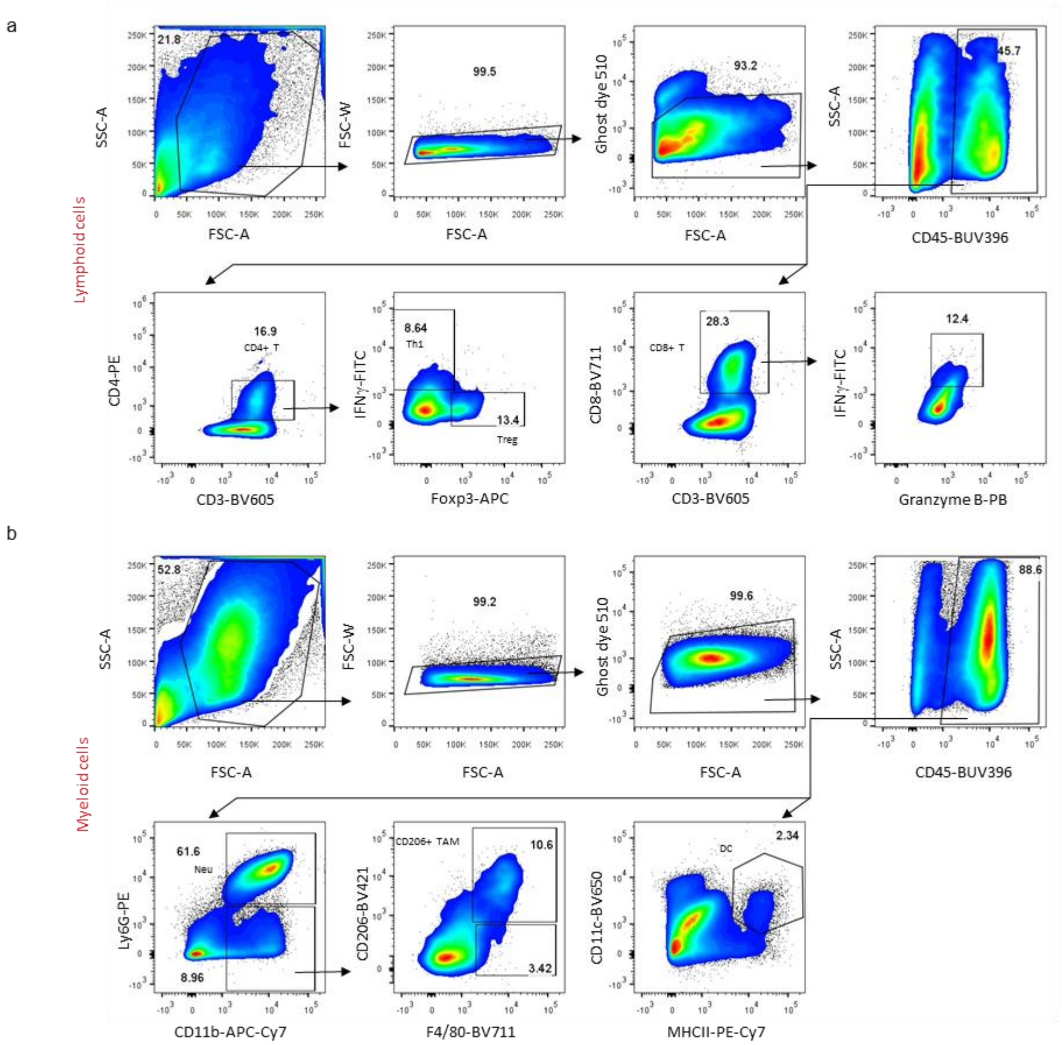

Supplementary Figure 9 | Flow cytometry gating strategy. a and b, Tumor gating strategy for lymphoid cells (**a**) and myeloid cells (**b**) in Figure 5d-j, 5p-r, and supplementary Figure 5g, 5h and 5j.

Supplementary Figure 10

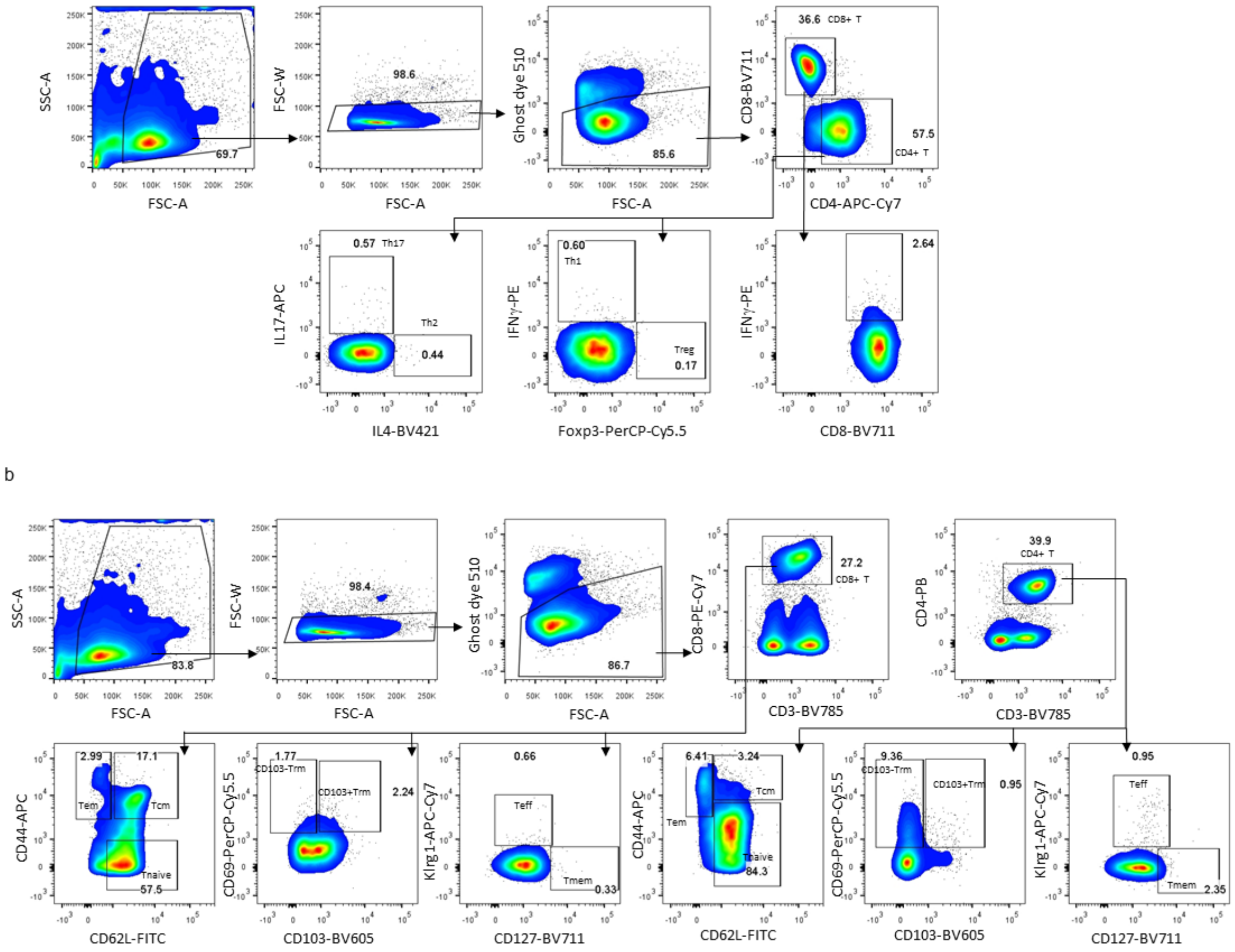

Supplementary Figure 10 | Flow cytometry gating strategy. a, Gating strategy for Th1, Th2, Th17, Treg and IFN-γ^{+}CD8^{+} T cells in Figure 6d, 6e, and Supplementary Figure 6a-c. **b,** Gating strategy for memory T cells in Figure 6f, 6h, 6j, Supplementary Figure 6d and 6f.

Supplementary Tables

Supplementary Table 1 | Clinicopathologic characteristics of the HCC patients receiving monotherapy with the anti-PD-1 antibody nivolumab

	n	
**Characteristic**	**Responders (*n* = 5)**	**Nonresponders (*n* = 5)**
Mean (± SEM) age, years	53.00 ± 4.95	53.40 ± 3.04
Sex		
Female	0	0
Male	5	5
AFP level		
≤20 ng/ml	5	1
>20 ng/ml	0	4
Liver cirrhosis		
No or mild	4	2
Moderate or severe	1	3
TNM stage		
I or II	5	2
III or IV	0	3
Vascular invasion		
No	5	4

Yes	0	1
Median (±) progression-free survival duration, months	11.54 ± 2.18	3.37 ± 1.29
12-month progression-free survival rate, %	60	0

Supplementary Table 2 | The most significantly upregulated and downregulated secreted proteins in nivolumab nonresponders among HCC patients (*P* < 0.001)

**Protein name**	**Log_2fold change**	***P* value**	**False-discovery rate**
DKK1	8.165195	1.65E-08	0.000320
HAPLN1	12.331900	2.58E-08	0.001306
TFF1	12.340000	5.62E-07	0.001316
MUC5B	12.374070	6.88E-07	0.001523
SEMA3E	8.011808	8.58E-07	0.003105
MMP9	4.200044	3.12E-06	0.005013
PCSK1N	7.706546	8.87E-06	0.005403
TCN1	10.35162	1.05E-05	0.005691
AFP	8.268480	1.21E-05	0.008479
DMBT1	11.372530	2.08E-05	0.013235
QPCT	4.779799	4.24E-05	0.014463
NPW	3.106201	4.89E-05	0.019218
SPOCK2	3.984415	8.11E-05	0.022359
TINAG	6.017802	0.000114	0.023700
NXPH4	4.915524	0.000125	0.023711
RNASE1	2.160843	0.000129	0.024350
TFF2	9.007115	0.000136	0.027357
S100A9	5.199195	0.000167	0.030233
OLFM4	9.232308	0.000207	0.030774

FGF4	11.815260	0.000217	0.036745
SOSTDC1	6.065715	0.000296	0.037738
MUC5AC	8.777202	0.000314	0.037928
REG3A	10.042110	0.000317	0.038289
NPTX2	5.596963	0.000337	0.041427
TFF3	3.467814	0.000368	0.043010
PRSS2	9.614341	0.000386	0.044438
MMP7	4.435444	0.000413	0.045538
EMILIN2	3.029353	0.000432	0.048805
C1QTNF3	4.949585	0.000483	0.050004
FREM1	6.313422	0.000507	0.057315
GLDN	4.061464	0.000682	0.062229
EDIL3	3.473021	0.000799	0.065790
FDCSP	6.437370	0.000897	0.067480
IHH	3.327172	0.000945	0.069083
DEFB1	-3.378010	0.000995	0.010813
ADAMTS17	-3.291470	0.000030	0.011545
PRG4	-3.079460	0.000033	0.024990
COL5A3	-3.657890	0.000142	0.026033
COL4A3	-4.341230	0.000152	0.031123
ANGPTL4	-2.649000	0.000220	0.044578
AFM	-2.853390	0.000418	0.050360
GDNF	-5.693550	0.000520	0.057315

CXCL2	-2.652790	0.000682	0.065000
FAM198A	-3.244090	0.000879	0.000320

Data were analyzed using two-sided t-test.

Supplementary Table 3. The association between RNase1 expression and clinicopathological characteristics of HCC patients.

Characteristics		Patient Number (%)	RNase1 expression		*P* value
			low (n = 84)	high (n = 90)	
Age, years	≤ 50	57 (28.2)	29	28	0.230
	> 50	117 (71.8)	55	62	
Gender	Female	21 (12.7)	13	8	1.777
	Male	153 (87.3)	71	82	
HBsAg	Negative	21 (13.6)	10	11	**0.004**
	Positive	153 (86.4)	84	79	
HCV	Negative	169 (45.5)	83	86	1.648
	Positive	5 (54.4)	1	4	
AFP, ng/ml	≤ 20	79 (45.5)	36	43	0.424
	> 20	95 (54.4)	48	47	
CEA, ng/ml	≤ 5	155 (87.3)	75	80	**0.007**
	> 5	19 (12.7)	9	10	
CA19-9, U/ml	≤ 36	143 (87.3)	71	72	0.925
	> 36	30 (12.7)	12	18	
GGT	≤ 54	96 (87.3)	47	49	**0.040**
	> 54	78 (12.7)	37	41	
Liver cirrhosis	No	37 (22.7)	20	17	0.628
	Yes	137 (77.3)	64	73	
Tumor number	Single	143 (82.7)	68	75	0.168
	Multiple	31 (17.3)	16	15	
Tumor size, cm	≤ 5	97 (59.1)	46	51	0.064
	> 5	77 (40.9)	38	39	
Tumor encapsulation	Complete	113 (71.8)	54	59	**0.031**
	None	61 (28.2)	30	31	
Tumor differentiation	I–II	112 (60.9)	53	59	0.115

	III–IV	62 (39.1)	31	31	
Microvascular invasion	Absent	116 (73.6)	60	56	1.657
	Present	58 (26.4)	24	34	
ALT, U/L	≤ 40	116 (65.5)	58	58	0.414
	> 40	58 (34.5)	26	32	
BCLC stage	0 + A	95 (60.0)	52	43	3.498
	B + C	79 (40.0)	32	47	
TNM stage	I + II	138 (81.8)	69	69	0.794
	III + IV	36 (18.2)	15	21	
Mean OS (months)			51.16±1.87	40.07±2.28	0.0004

Abbreviations: HCC, hepatocellular carcinoma; HBsAg, hepatitis B surface antigen; HCV, hepatitis C virus; AFP, α-fetoprotein; CEA, carcinoembryonic antigen; CA19-9, carbohydrate antigen 19-9; GGT, Gamma-glutamyl transferase; ALT, alanine transaminase; BCLC, Barcelona Clinic Liver Cancer; TNM, tumor-nodes-metastasis.

P values were calculated using the two-sided Pearson chi-square test. **P* value of < 0.05 was considered statistically significant. Bold *P* values indicate statistical significance.

Supplementary Table 4. Univariate and multivariate analyses of factors associated with survival and recurrence in HCC cohort.

	OS			RFS		
	**Univariate**	**Multivariate**		**Univariate**	**Multivariate**	
**Variables**	***P* Value**	**HR (95 % CI)**	***P* value**	***P* value**	**HR (95 % CI)**	***P value***
Age, years (> 50 vs. ≤ 50)	0.637		NA	0.848		NA
Gender (male vs. female)	0.900		NA	0.421		NA
HBsAg (positive vs. negative)	0.468		NA	0.527		NA
AFP, ng/ml (> 20 vs. ≤ 20)	0.302		NA	0.688		NA
CEA, ng/ml (> 5 vs. ≤ 5)	0.675		NA	0.487		NA
CA19-9, U/ml (> 36 vs. ≤ 36)	0.054		NA	0.222		NA
Ascites (present vs. absent)	0.202		NA	0.954		NA
Liver cirrhosis (yes vs. no)	**0.019**	**2.078(1.105-3.910)**	**0.023**	**0.030**	**1.943(1.041-3.625)**	**0.037**
Tumor number (multiple vs. single)	0.301		NA	0.377		NA
Tumor size, cm (> 5 vs. ≤ 5)	**0.038**	1.519(0.931-2.477)	0.094	0.551		NA
Tumor encapsulation (complete vs. none)	**0.028**	1.459(0.930-2.288)	0.100	0.306		NA
BCLC stage (B+C vs 0+A)	**0.003**	0.983(0.405-2.387)	0.969	**0.044**	1.331(0.643-2.757)	0.441
Microvascular invasion (present vs. absent)	**0.021**	1.265(0.599-2.670)	0.538	**0.039**	0.877(0.410-1.873)	0.734
ALT, U/L (> 40 vs. ≤ 40)	0.239		NA	0.572		NA
TNM stage (III-IV vs I-II)	**0.001**	1.399(0.691-2.834)	0.351	0.698		NA
Child grade (B+C vs A)	0.400		NA	0.174		NA
RNase1 (high vs. low)	**0.001**	**1.994(1.280-3.105)**	**0.002**	**0.035**	**1.573(1.002-2.470)**	**0.049**

Data obtained from the Cox proportional hazards model (two-sided Likelihood ratio test). *P* value <0.05 was regarded as statistically significant.

Abbreviations: OS, overall survival; RFS recurrence-free survival; HBsAg, hepatitis B surface antigen; AFP: α-fetoprotein; CEA: carcinoembryonic antigen; CA19-9: carbohydrate antigen 19-9; ALT: alanine transaminase; BCLC, Barcelona Clinic Liver

Cancer; TNM, tumor-nodes-metastasis; HR, hazard ratio; CI, confidential interval; NA, not adopted.

Supplementary Table 5. Clinicopathologic characteristics of PD-1 mAb monotherapy cohort (n=13).

	PD-1 mAb Monotherapy	
Characteristics	Responders<br>n = 5	Non-responders<br>n = 8
Age (years), mean ± SEM	49.2±3.26	55.63±3.88
Gender		
Female	1	1
Male	4	7
AFP level (ng/ml)		
≤ 20	1	2
> 20	4	6
Liver cirrhosis		
No or mild	2	5
Moderate or Severe	3	3
Vascular invasion		
Without	2	2
With	3	6
Alanine transaminase (U/L)		
≤ 40	2	5
> 40	3	3
CEA (ng/ml)		
≤ 5	4	8
> 5	1	0
TNM stage		
I or II	5	8
III or IV	0	0

Median PFS (months)	18.19±10.37	2.47±0.57
12 months PFS (%)	40	0

Supplementary Table 6 | Antibodies used for cytometry by time of flight (provided by Fluidigm)

Marker	Clone	Label	Dilution
CD3e	145-2C11	152Sm	1:100
CD4	RM4-5	145Nd	1:200
CD8a	53-6.7	168Er	1:200
CD11c	Polyclonal	142Nd	1:100
CD11b	M1/70	148Nd	1:200
Ly6G	1A8	141Pr	1:200
CD19	6D5	149S	1:200
F4/80	BM8	159Tb	1:200
Ly6C	HK1.4	150Nd	1:200

Supplementary Table 7 | Correlation between RNase1 level and immune cell infiltration

**Immune cells**	**Correlation coefficient (*R*)**	***P* value**
Macrophages	0.46	6.36e-20
DCs	0.27	2.65e-07
Eosinophils	-0.21	5.77e-05
Mast cells	0.24	4.79e-06
Neutrophils	0.23	1.22e-05
CD56bright NK cells	0.23	7.95e-06
CD56dim NK cells	0.23	1.10e-05
NK cells	0.03	5.38e-01
αDCs	0.27	2.23e-07
B cells	0.02	6.69e-01
CD8 T cells	0.04	4.99e-01
Cytotoxic cells	0.20	1.32e-04
iDCs	0.45	3.70e-19
pDCs	0.10	6.07e-02
T cells	0.25	2.07e-06
T helper cells	0.13	1.54e-02
Tcm cells	-0.21	4.42e-05
Tem cells	0.21	7.59e-05
TFH cells	0.21	6.78e-05
Tgd cells	-0.04	4.88e-01
Th1 cells	0.33	9.39e-11

Th17 cells	-0.23	1.47e-05
Th2 cells	0.19	3.34e-04
Tregs	-0.01	8.67e-01

Data were analyzed using the two-tailed statistical significance of Pearson's correlation coefficient.

Supplementary Table 8 | RNase1 expression for response and non-response based on pre-treatment samples in each dataset

Study#	Cancers	Anti Target	Response Mean	Non-Response Mean	Log_2FC	FDR	*P* Value	n
ERP107734	Gastric Cancer	anti-PD1	3,910.00	7,124.00	-0.921	0.228	0.045	45
SRP128156	RCC	anti-PD1/anti-PD1+anti-CTLA4	1,088.00	1,514.00	1.434	0.593	0.010	29

Data were analyzed using two-sided t-test.

Supplementary Table 9 | Antibodies used for flow cytometry

Antibody	Clone	Catalog number	Company	Dilution
CD206-BV421	C068C2	141717	Biolegend	1:200
iNOS-PE	CXNFT	12-5920-80	Invitrogen	1:200
F4/80-BV711	BM8	123147	Biolegend	1:200
CD11b-PerCp-Cy5.5	M1/70	550993	BD	1:400
Ly6G-FITC	1A8	11-9668-82	eBioscience	1:400
CD11c-PE-Cy7	N418	117318	Biolegend	1:200
CD11b-PerCp-Cy5.5	M1/70	550993	BD	1:400
CD4-APC-Cy7	GK1.5	552051	BD	1:200
CD8-BV711	53-6.7	100748	Biolegend	1:200
IFN-γ-PE	XMG1.2	12-7311-41	Invitrogen	1:200
Foxp3-PerCp-Cy5.5	FJK-16s	45-5773-82	Invitrogen	1:200
IL-17A-Alexa Fluor 647	TC11-18H10	560184	BD	1:200
IL-4-BV421	11B11	562915	BD	1:200
Granzyme B-Pacific blue	GB11	515408	Biolegend	1:50
IFN-γ-FITC	XMG1.2	11-7311-82	eBioscience	1:200
CD4-EF450	GK1.5	48-0041-82	Invitrogen	1:200
CD8-PE-Cy7	53-6.7	60-0081-U100	Tonbo	1:200
CD3-BV785	17A2	100232	Biolegend	1:50
CD103-BV605	2E7	121433	Biolegend	1:200

CD44-APC	1M7	103012	Biolegend	1:200
CD62L-FITC	MEL-14	104406	Biolegend	1:200
CD69-PerCp-Cy5.5	H1.2F3	45-0691-82	eBioscience	1:200
CD127-BV711	A7R34	135035	Biolegend	1:50
Klrg1-APC-Cy7	2F1	138426	Biolegend	1:100
Ghost dye-Violet 510	NA	13-0870-1100	Tonbo	1:300
