# Supplementary material for: Targeting ALK averts ribonuclease 1-induced immunosuppression and enhances antitumor immunity in hepatocellular carcinoma
Source: Nat Commun. 2024 Feb 2;15:1009. doi: 10.1038/s41467-024-45215-0 (PMC10837126; doi:10.1038/s41467-024-45215-0)
Supplement: Supplementary file 3 — Reporting Summary [file 41467_2024_45215_MOESM3_ESM.pdf]

nature portfolio

Corresponding author(s): Mien-Chie Hung

Last updated by author(s): 1/15/2024

Reporting Summary

Nature Portfolio wishes to improve the reproducibility of the work that we publish. This form provides structure for consistency and transparency in reporting. For further information on Nature Portfolio policies, see our Editorial Policies and the Editorial Policy Checklist.

Statistics

For all statistical analyses, confirm that the following items are present in the figure legend, table legend, main text, or Methods section.

n/a	Confirmed	
☐	☒	The exact sample size (*n*) for each experimental group/condition, given as a discrete number and unit of measurement
☐	☒	A statement on whether measurements were taken from distinct samples or whether the same sample was measured repeatedly
☐	☒	The statistical test(s) used AND whether they are one- or two-sided<br>*Only common tests should be described solely by name; describe more complex techniques in the Methods section.*
☒	☐	A description of all covariates tested
☐	☒	A description of any assumptions or corrections, such as tests of normality and adjustment for multiple comparisons
☐	☒	A full description of the statistical parameters including central tendency (e.g. means) or other basic estimates (e.g. regression coefficient) AND variation (e.g. standard deviation) or associated estimates of uncertainty (e.g. confidence intervals)
☐	☒	For null hypothesis testing, the test statistic (e.g. *F*, *t*, *r*) with confidence intervals, effect sizes, degrees of freedom and *P* value noted<br>*Give P values as exact values whenever suitable.*
☒	☐	For Bayesian analysis, information on the choice of priors and Markov chain Monte Carlo settings
☒	☐	For hierarchical and complex designs, identification of the appropriate level for tests and full reporting of outcomes
☒	☐	Estimates of effect sizes (e.g. Cohen's *d*, Pearson's *r*), indicating how they were calculated

Our web collection on statistics for biologists contains articles on many of the points above.

Software and code

Policy information about availability of computer code

Data collection	Flow cytometry data was acquired using FACSDiva software (v8.0).
Data analysis	Statistical analyses were performed using GraphPad Prism program (v8), including two-tailed Student's t-test, ordinary one-way ANOVA, and ordinary two-way ANOVA.<br>Flow cytometry files (.fcs) were analyzed using FlowJo software (v10.6.2).

For manuscripts utilizing custom algorithms or software that are central to the research but not yet described in published literature, software must be made available to editors and reviewers. We strongly encourage code deposition in a community repository (e.g. GitHub). See the Nature Portfolio guidelines for submitting code & software for further information.

Data

Policy information about availability of data

All manuscripts must include a data availability statement. This statement should provide the following information, where applicable:

- Accession codes, unique identifiers, or web links for publicly available datasets
- A description of any restrictions on data availability
- For clinical datasets or third party data, please ensure that the statement adheres to our policy

RNA-sequencing expression profiles and clinical information for TCGA HCC patients are publicly available and downloaded from the TCGA database (https://portal.gdc.cancer.gov/). The relevance of gene mRNA level to OS of pan-cancer are publicly available and downloaded from Kaplan-Meier plotter database (http://

kmplot.com/analysis/). Immunologic signature gene sets are publicly available and downloaded from the MSigDB database (https://www.gsea-msigdb.org/gsea/msigdb). The immune checkpoint blockade therapy data for gastric cancer (ERP107734) and renal cell carcinoma (RCC) (SRP128156) are publicly available from ICBatlas database (http://bioinfo.life.hust.edu.cn/ICBatlas/#!/). The RNA-seq data for 10 HCC patients and THP-1 cells that used in this study are available in the Gene Expression Omnibus database under accession code GSE215011 and GSE215012. The remaining data are available within the Article, Supplementary Information or Source Data file. Source data are provided with this paper.

Human research participants

Policy information about studies involving human research participants and Sex and Gender in Research.

Reporting on sex and gender	The samples used in this study are from both female and male patients.
Population characteristics	HCC tumor tissue samples from immunotherapy cohorts, Human HCC tissue microarray samples, and plasma samples were obtained from Zhongshan Hospital, Fudan University, Shanghai, China. The use of human HCC samples and the relevant database was approved by the Zhongshan Hospital Research Ethics Committee and complied with all relevant ethical regulations. All tissue samples were collected in compliance with informed consent policy. A total of 23 patients who were standard treated with anti-PD-1 antibodies between May 2020 and January 2021 included in this study. A total of 174 patients undertook surgical resection, and 67 of 174 patients' plasma samples were collected to do ELISA experiments. Detail Clinicopathologic characteristics of those patients are provided in supplemental table 1, 3 and 5. The staining of the tissue sections was quantitatively scored according to the percentage of positive cells and staining intensity. Scores were compared with overall survival duration, defined as the time from date of diagnosis to that of death or last known follow-up examination.
Recruitment	participants were not recruited.
Ethics oversight	Zhongshan Hospital Institutional Review Board

Note that full information on the approval of the study protocol must also be provided in the manuscript.

Field-specific reporting

Please select the one below that is the best fit for your research. If you are not sure, read the appropriate sections before making your selection.

[x] Life sciences [ ] Behavioural & social sciences [ ] Ecological, evolutionary & environmental sciences

For a reference copy of the document with all sections, see nature.com/documents/nr-reporting-summary-flat.pdf

Life sciences study design

All studies must disclose on these points even when the disclosure is negative.

Sample size	The group sizes of the animals chosen are based on the numbers we used for previous publications, which is most optimal to generate statistically significant results and to provide a sufficient level of statistical power for detecting indicated biological effects.
Data exclusions	No data were excluded.
Replication	Experimental findings were confirmed by performing 2-5 independent experiments as indicated in the figure legends. Biological replicates and independent experiments were used to ensure reproducibility of results.
Randomization	For in vivo mouse model, mice were chosen randomly for inclusion in different treatment groups. For other experiments, the samples/cells for were randomized to be examined (No specific methods were used).
Blinding	IHC analysis was performed blindly. For other experiments, blinding is not relevant because all experiment groups were conducted with different treatments.

Reporting for specific materials, systems and methods

We require information from authors about some types of materials, experimental systems and methods used in many studies. Here, indicate whether each material, system or method listed is relevant to your study. If you are not sure if a list item applies to your research, read the appropriate section before selecting a response.

Materials & experimental systems

n/a	Involved in the study	
☐	☒	Antibodies
☐	☒	Eukaryotic cell lines
☒	☐	Palaeontology and archaeology
☐	☒	Animals and other organisms
☒	☐	Clinical data
☒	☐	Dual use research of concern

Methods

n/a	Involved in the study	
☒	☐	ChIP-seq
☐	☒	Flow cytometry
☒	☐	MRI-based neuroimaging

Antibodies

Antibodies used	The primary antibodies used for Western blotting, immunofluorescent analysis, and immunoprecipitation were rabbit anti-RNase1 (Polyclonal, cat. no. HPA001140; Atlas Antibodies; 1:1000) and mouse anti-tubulin (clone B-5-1-2, cat. no. T5168; Sigma-Aldrich; 1:3000); rabbit anti-phospho-ALK (Y1057, cat. no. ab192809; Abcam; 1:1000) and rat anti-CD8 (clone YTS169.4, cat. no. ab22378; Abcam; 1:100); mouse anti-EphA4 (clone M280, cat. no. EM2801; ECM biosciences; 1:1000) and anti-phospho-EphA4 (Y602, cat. no. EP2731; ECM Biosciences; 1:1000); rabbit anti-PD-L1 (clone 2096C, cat. no. MAB90781; R&D Systems; 1:100) and goat anti-granzyme B (cat. no. AF1865; R&D Systems; 1:100); mouse anti-ALK (clone F-12, cat. no. sc-398791; Santa Cruz Biotechnology; 1:1000) and mouse anti-STAT3 (clone F-2, cat. no. SC-8019; Santa Cruz Biotechnology; 1:2000); rabbit anti-phospho-STAT3 Tyr705 (clone D3A7, cat. no. 9145S; Cell Signaling Technology; 1:2000), rabbit anti-phospho-STAT1 (clone 58D6, cat. no. 9167; Cell Signaling Technology; 1:2000), rabbit anti-phospho-ERK1/2 Thr202/Tyr204 (Polyclonal, cat. no. 9101; Cell Signaling Technology; 1:3000), rabbit anti-ERK1/2 (Polyclonal, cat. no. 9102; Cell Signaling Technology; 1:3000), and rabbit anti-iNOS (Polyclonal, cat. no. 2977; Cell Signaling Technology; 1:1000); and rabbit anti-CD206 (Polyclonal, cat. no. NBP1-90020; Novus Biologicals; 1:100). The following antibodies were used for immunohistochemical analyses: goat anti-PD-L1 (Polyclonal, cat. no. AF1019; R&D Systems; 1:200), rabbit anti-RNase1 (Polyclonal, cat. no. HPA001140; Sigma-Aldrich; 1:500), rabbit anti-CD206 (Polyclonal, cat. no. NBP1-90020; Novus Biologicals; 1:500), rabbit anti-granzyme B (Polyclonal, cat. no. 4059; Abcam; 1:200), rabbit anti-phospho-ALK (Polyclonal, cat. no. 3341; Cell Signaling Technology; 1:100), rabbit anti-CD8 (clone SP16, cat. no. ab101500; Abcam; 1:100), and rabbit anti-CD68 (clone EPR20545, cat. no. ab213363; Abcam; 1:200). The following antibodies were used for the animal studies: 100g of a mouse anti-PD-1 antibody (cat. no. BE0146) and IgG control (cat. no. BE0089; Bio X Cell).<br><br>Antibodies used for CyTOF and flow cytometry were listed Supplementary Table 6 and 9.
Validation	Antibodies were only used for the application as indicated and organisms verified by the manufactures.

Eukaryotic cell lines

Policy information about cell lines and Sex and Gender in Research

Cell line source(s)	HEK 293T cells (CRL-3216), Hep3B cells (HB-8064), HepG2 (HB-8065), Hepa1-6 (CRL-1830) and PLC/PRF/5 (CRL-8024) were purchased from American Type Culture Collection (ATCC). HCC cell lines Huh7, HCA-1, HA22T, HA59T, Tong, and Mahlavu were obtained from the China Medical University Hospital. The cell lines used in this study were authenticated by STR profiling and tested for the absence of mycoplasma contamination.
Authentication	Cells were authenticated by short tandem repeat DNA finger printing at The University of Texas MD Anderson Cancer Center (Houston, Texas)
Mycoplasma contamination	Cells are negative for mycroplasma.
Commonly misidentified lines<br>(See ICLAC register)	No commonly misidentified lines were used.

Animals and other research organisms

Policy information about studies involving animals; ARRIVE guidelines recommended for reporting animal research, and Sex and Gender in Research

Laboratory animals	C3H and C57BL/6 mice (male, 6-8 weeks old; The Jackson Laboratory) were used. All mice were housed in 12 hours light/dark cycle with controlled room temperature (23±2°C) and humidity (30-70%).
Wild animals	This study did not involve wild animals
Reporting on sex	Male
Field-collected samples	No sample was collected from the field.
Ethics oversight	The use of the animals was approved by the Institutional Review Board and the Institutional Animal Care and Use Committee (IACUC) of The university of Texas MD Anderson Cancer Center

Note that full information on the approval of the study protocol must also be provided in the manuscript.

Flow Cytometry

Plots

Confirm that:

- [x] The axis labels state the marker and fluorochrome used (e.g. CD4-FITC).
- [x] The axis scales are clearly visible. Include numbers along axes only for bottom left plot of group (a 'group' is an analysis of identical markers).
- [x] All plots are contour plots with outliers or pseudocolor plots.
- [x] A numerical value for number of cells or percentage (with statistics) is provided.

Methodology

Sample preparation	For flow cytometric analysis, single-cell suspensions were generated from mouse spleens and TdLNs by smashing these tissues with a sterile syringe plunger onto a cell strainer; cells were rinsed through the strainer with RPMI/2% fetal bovine serum at room temperature. Regarding the spleens, red blood cells were first removed using RBC Lysis Buffer (Tonbo Bioscience) following the manufacturer's recommendation. Tumors were digested using a mouse Tumor Dissociation Kit. Tumor-infiltrating lymphocytes were enriched on a Ficoll gradient (Sigma-Aldrich). Single-cell suspensions were then incubated with antibodies at 1 × 106 cells per sample. In all samples, cell surface Fc receptors were blocked via incubation with a rat anti-mouse CD16/32 antibody (1:100; Tonbo Bioscience) for 15 min at 4 °C. Cells were then incubated with fluorescently conjugated antibodies against surface markers for 30 min at 4 °C. For intracellular antibody staining, cells were fixed and permeabilized using commercial reagents (Intracellular Fixation and Permeabilization Buffer Set, eBioscience) and stained with antibodies against intracellular proteins for 30 min at 4 °C. All antibodies were purchased from BioLegend, eBioscience, BD Biosciences, or Tonbo Biosciences (Supplementary Table 5). Dead cells were discriminated in all experiments using Ghost Dye Violet 510 (Tonbo Biosciences).
Instrument	LSRII and LSRFortessa flow cytometers (BD)
Software	Data was acquired using FACSDiva 8.0 software (BD). Flow cytometry files (.fcs) were analyzed using FlowJo 10.6.2 software (BD)
Cell population abundance	NA
Gating strategy	lymphocytes were identified based on their forward scatter (FSC-A) and side scatter (SSC-A) profiles. Doublets and dead cells were excluded. Gating strategies of immune cells are shown in supplemental figure 8.

- [x] Tick this box to confirm that a figure exemplifying the gating strategy is provided in the Supplementary Information.
